# Factors Associated with Coverage of Cotrimoxazole Prophylaxis in HIV-Exposed Children in South Africa

**DOI:** 10.1371/journal.pone.0063273

**Published:** 2013-05-07

**Authors:** Dhayendre Moodley, Leanne Reddy, Wisani Mahungo, Rebotile Masha

**Affiliations:** Womens Health and HIV Research Unit, Department of Obstetrics and Gynaecology, Nelson R Mandela School of Medicine, University of KwaZulu–Natal, Durban, South Africa; University of Cape Town, South Africa

## Abstract

**Background:**

The World Health Organisation and the Joint United Nations Programme in 2006 reaffirmed the earlier recommendation of 2000 that all HIV-exposed infants in resource-poor countries should commence cotrimoxazole (CTX) prophylaxis at 6-weeks of life. CTX prophylaxis should be continued until the child is confirmed HIV-uninfected and there is no further exposure to breastmilk transmission. We determined CTX coverage and explored factors associated with CTX administration in HIV-exposed infants at a primary health clinic in South Africa.

**Methods:**

In a cross-sectional study of HIV-exposed infants 6–18 months of age attending a child immunisation clinic, data from the current visit and previous visits related to CTX prophylaxis, feeding practice and infant HIV testing were extracted from the child's immunisation record. Further information related to the administration of CTX prophylaxis was obtained from an interview with the child's mother.

**Results:**

One-third (33.0%) HIV-exposed infants had not initiated CTX at all and breastfed infants were more likely to have commenced CTX prophylaxis as compared to their non-breastfed counterparts (78.7% vs 63.4%) (p = 0.008). Availability of infant's HIV status was strongly associated with continuation or discontinuation of CTX after 6 months of age or after breastfeeding cessation. Maternal self-reports indicated that only 52.5% (95%CI 47.5–57.5) understood the reason for CTX prophylaxis, 126 (47%) did not dose during weekends; 55 (21%) dosed their infants 3 times a day and 70 (26%) dosed their infants twice daily.

**Conclusion:**

A third of HIV-exposed children attending a primary health care facility in this South African setting did not receive CTX prophylaxis. Not commencing CTX prophylaxis was strongly associated with infants not breastfeeding and unnecessary continued exposure to CTX in this paediatric population was due to limited availability of early infant diagnosis. Attendance at immunization clinics can be seen as missed opportunities for early infant diagnosis of HIV and related care.

## Introduction

Cotrimoxazole (CTX) prophylaxis has been known for its protective effect against opportunistic infections in adults and its benefits have also been demonstrated in HIV infected children [1], [2]. Hence the recommendation of the World Health Organisation (WHO) and the Joint United Nations Programme on HIV/AIDS (UNAIDS) in 2004, that all HIV exposed infants should receive CTX from 4–6 weeks of age until infants are confirmed HIV uninfected and no longer exposed to HIV through breastfeeding. Yet, only 4% of HIV-exposed and HIV infected children in sub-Saharan Africa were receiving CTX prophylaxis in 2007, 8% in low- and middle income countries in 2008 and 14% globally in 2009 [3]–[5].

The WHO/UNAIDS recommendations for universal CTX prophylaxis in all HIV exposed infants and HIV infected children were made when mother-to-child transmission (MTCT) rates were reportedly much higher in the absence of interventions to reduce the risk of MTCT. With more complex and efficacious antiretroviral (ART) prophylactic regimens being implemented globally to reduce the risk of transmission during pregnancy, labour and delivery and during breastfeeding, fewer children are expected to be HIV infected [6], [7]. KwaZulu-Natal in South Africa remains the epicentre of the HIV pandemic with an antenatal HIV prevalence of 39.1% and historical studies in predominantly breastfed study populations in this province revealed transmission rates of 34% at 12 months in the absence of any intervention (1996), 19% at 6 months if infants were exclusively breastfed (2000) and 12% at 8 weeks with the use of single dose Nevirapine (2003) [8]–[11]. In 2010, an evaluation of the South African PMTCT program that included maternal and infant dual ART prophylaxis (60% coverage), triple ART treatment among women with CD4<200 (34% coverage) and exclusive breastfeeding (24% coverage) revealed a MTCT rate of 4.0% (95%CI 3.3%–4.8%) at 8 weeks of age [12]. Considering the lower MTCT rate achieved in recent years, less than 5% of HIV exposed children would essentially benefit from CTX prophylaxis although 95% would require it until breastfeeding cessation and confirmation of their negative HIV status. Estimating coverage of CTX prophylaxis for HIV exposed infants as reported by WHO and UNAIDS would therefore be dependent on the availability of early infant diagnosis and infant feeding norms in a particular setting.

We examined coverage of CTX prophylaxis in association with early infant diagnosis and infant feeding practices at a primary health clinic in KwaZulu Natal, South Africa.

## Methods

In a study of a cross-sectional sample of children aged 6≤18 months, we determined the proportion of HIV exposed infants that initiated CTX prophylaxis. We also describe how infant feeding practice and early infant diagnosis influence the implementation of the WHO/UNAIDS guidelines on infant CTX prophylaxis.

Routine childhood immunisation provided at the primary health care facilities include DTP, Polio, Hib and HepB vaccines at 6, 10 and 14 weeks of age, Measles at 9 months of age and Polio, DTP and Measles at 18 months of age. Children brought to the immunisation services at a primary health clinic in Umlazi, KwaZulu Natal between May 2009 and August 2010 were screened for study selection. Umlazi, an urban subdistrict in Ethekwini (Durban) with an antenatal HIV prevalence of 42% (2010) is the second largest township in South Africa and offers a health service to its community through 17 primary health clinics. The clinic chosen for the study is one of 3 primary health centres with the highest child immunization attendance in the Umlazi subdistrict.

HIV positive mothers of 6 to 18 month old infantswere approached to participate in the study. Infants were excluded from the study if their mothers refused participation, or if they did not have a Child Health Record or if the mother's HIV status was negative or not known. At the time of the study, HIV positive women at this clinic were provided free replacement feeds if they chose to formula feed or advised to exclusively breastfeed for 4 to 6 months if they chose to breastfeed. HIV exposed infants were tested for HIV by DNA PCR and the laboratory results if available were discussed with the mother at the next scheduled immunisation visit. The clinic followed the 2008 PMTCT guidelines that included the WHO recommendations of CTX prophylaxis in HIV exposed infants [13].

A structured questionnaire was administered by a trained research assistant and mothers of the infants were asked of her knowledge of CTX prophylaxis and how she had administered CTX to her infant. The child's Health Record was reviewed and the infant HIV status, information on infant feeding practice and evidence of CTX dispensation for all visits prior to and including the current visit, were extracted.

Data were captured in Epi-Info Version 2.0. Each participant had a unique identifier and the relevant information collected at the index visit (real–time) as well as all previous visits (retrospective) were captured per participant. The statistical analysis was performed using Stata version 2.0 (StataCorp, Texas, USA) and where applicable a descriptive analysis included the means, medians, range and 95%CI. Comparisons were made using the Pearson's chi-square test in the case of categorical data and associations were considered statistical significant at the 95% level (p<0.05).

A written informed consent was obtained from the mothers of HIV exposed children who were eligible for participation in the study. The protocol and the informed consent were approved by the University of KwaZulu Natal Ethics Committee and the KwaZulu Natal Department of Health.

## Results

During the period May 2009 to August 2010, 400 HIV exposed children ranging between 6–18 months of age were included in the study and analysed in 3 categorical age groups, 6–9 months of age (61%%), 10–12 months of age (24%) and between 13 and 18 months of age (15%). The median age of the HIV exposed infants at the time of the cross-sectional analysis was 9.0 months. Three hundred and 5 (76.5%) of these infants were never breastfed since birth; and the mean age of breastfeeding cessation among the 95 infants (23.5%) who were breastfed was 5.6 months (range 1 week–12 months).

The HIV status was known for 69.7% of the children at the time of evaluation; 3.5% were confirmed HIV positive by PCR and 66.3% were HIV negative. The HIV status was unknown for 32.4%, 21.9% and 35.0% of the children evaluated in the 6–9 month, 10–12 month and 13–18 month age categories respectively. The median number of clinic visits was 7 visits for the entire study population, with 141 (35%) infants with recorded visits less than the minimum required clinic visits. (Table 1).

**Table 1:** Characteristics of Infants enrolled in the Evaluation.

Age Groups		6–9 months	10–12 months	13–18 months	Total
**Number**		244	96	60	400
**HIV Status As At Evaluation Visit (PCR) % (95%CI)**	Positive	3.7(1.7–6.9)	2.1(0.3–7.3)	5.0(1.0–13.9)	14 (3.5%)
	Unknown	32.4(26.5–38.6)	21.9(14.1–31.5)	35.0(23.1–48.4)	121 (30.3%)
**Initiated CTX**	Yes	159 (65.2%)	61 (63.5%)	48 (80.0%)	268(67.0%)
**N(%)**	No	85(34.8%)	35 (36.5%)	12 (20.0%)	132(33.0%)
**Ever Breastfed**	Yes	58 (23.8%)	21(21.9%)	16 (26.7%)	95 (23.7%)
**N(%)**	No	186(76.2%)	75 (78.1%)	44 (73.3%)	305(76.3%)
**Number of Clinic Visits**	Median (IQR)	6 (1–8)	8 (6–10)	8 (1–11)	N/A
**Required Number of Immunisation Visits**	N	4	5	6	N/A
**Infants who attended less than Required Number of Immunisation Visits**		94 (38.5%)	19 (19.8%)	28 (46.7%)	N/A

**Figure 1:**
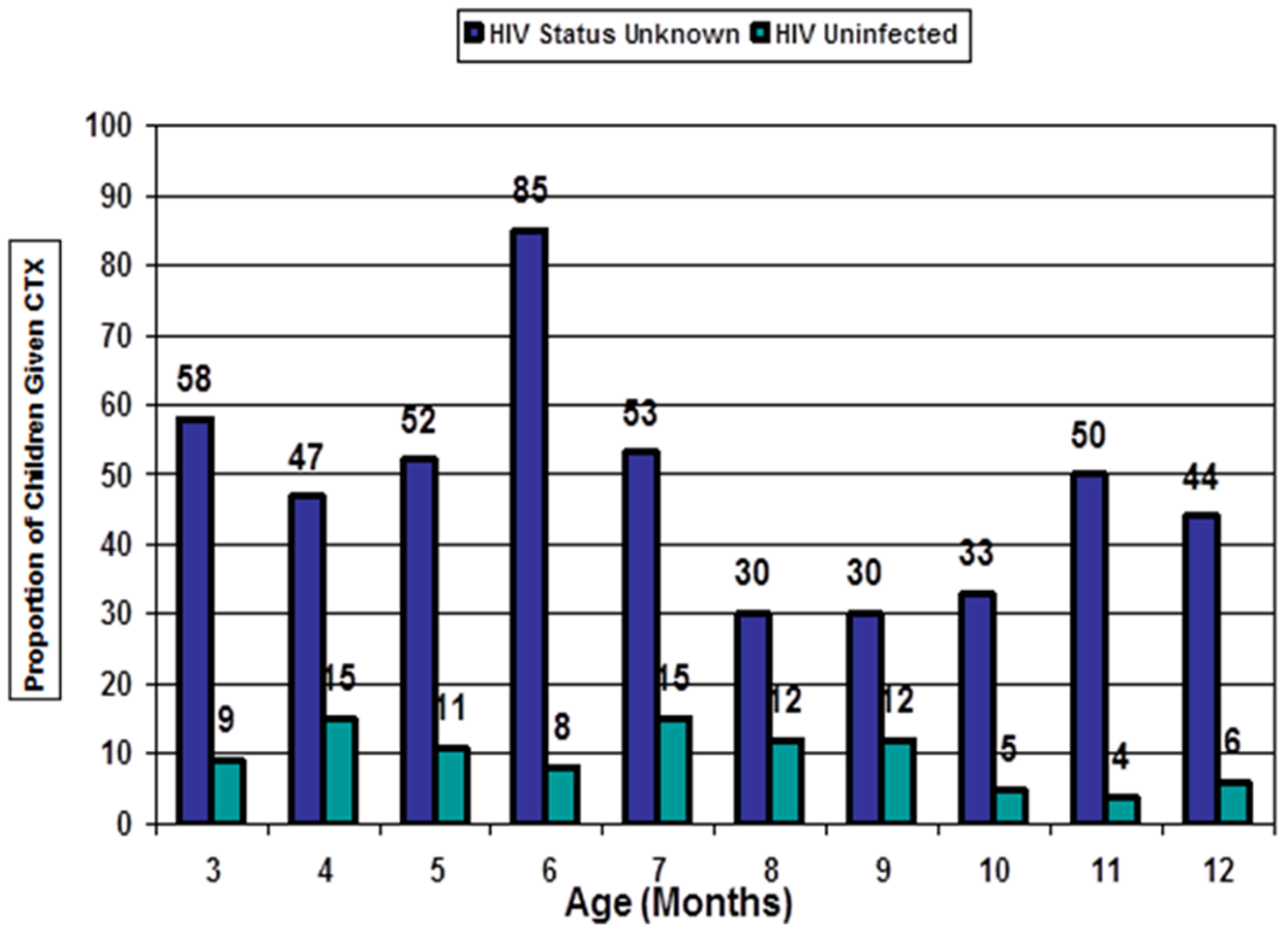
Coverage of cotrimoxazole prophylaxis in infants in association with HIV infection status. In this predominantly non-breastfed population, infants in the various age categories were still receiving cotrimoxazole prophylaxis despite their known “HIV-Uninfected Status” as represented by the green column. A larger proportion of infants were also continuing cotrimoxazole prophylaxis across the increasing age categories, due to their “Unknown HIV Status” as represented by the blue column.

One hundred and thirty two (33.0%; 95%CI 28.7–38.1) infants 6 months and older at the time of the evaluation had never initiated CTX prophylaxis at their prior visits. A significantly higher proportion of breastfed children initiated CTX prophylaxis at 6 weeks of age as compared to infants who were never breastfed (78.7% vs 63.4%; p = 0.006). HIV infected children (92.9%) and children whose HIV status was not known (71.4%) were more likely to have initiated CTX as compared to children who were HIV uninfected (64%) (p<0.05). Availability of the infant's HIV status was also strongly associated with continuing or discontinuing CTX prophylaxis particularly after 6 months of age or after cessation of breastfeeding (Figure 1).

Among the infants who initiated CTX, 251 (93.7%) of the mothers reported that the clinic dispensed adequate supply of CTX until the next scheduled visit. Half (52.5% 95%CI 47.5–57.5) of the 400 mothers understood the reason for CTX prophylaxis, 126 (47%) did not dose during weekends; 55 (21%) dosed their infants 3 times a day and 70 (26%) dosed their infants twice daily.

## Discussion

Two-thirds (67%) of the HIV exposed children in our study population had initiated CTX prophylaxis, and breastfed infants were more likely to have received CTX from 6 weeks. Although exclusive breastfeeding for 6 months is actively promoted for HIV positive pregnant South African women, it is not surprising that 75% of the study population had chosen not to breastfeed given the option of a free supply of formula milk for a period of 6 months [13]. Although all children, independent of infant feeding choice, should have commenced CTX prophylaxis at 6 weeks, our analysis reveals that infants who were never breastfed were more likely not to have initiated CTX at 6 weeks.

In countries where earlier and more reliable diagnostic services are available and women choose not to breastfeed or breastfeed for an average of 6 months, CTX prophylaxis in all HIV exposed children is expected to be initiated at 6 weeks of age independent of the feeding method. If the WHO recommendations are followed, CTX prophylaxis is to be provided until the infant is 8 to 9 months of age if infant is breastfed or 10 to 14 weeks of age if infant was never breastfed [3].

Recent revisions to the WHO/UNAIDS/UNICEF infant feeding recommendations and changes in the local South African policy in providing free formula milk could potentially influence the provision of CTX prophylaxis in the country [14], [15]. Women who do not meet the AFASS (Acceptable, Feasible, Affordable, Sustainable and Safe) criteria in the first 6 months and choose to breastfeed are likely not to meet these criteria beyond 6 months and as a result of effective counselling women will breastfeed for longer. Despite a potential improvement in the implementation of early infant diagnosis over time, CTX prophylaxis would still have to be provided for a longer period (12 months or more) until breastfeeding ceases.

In our evaluation, CTX prophylaxis in the breastfed infants continued beyond 7 to 8 months and well after cessation of breastfeeding. Not knowing the HIV status of the infants emerged as the primary indication for continued CTX prophylaxis in this group. Confirming HIV status in HIV exposed children younger than 15 months of age remains a challenge in resource limited countries if PCR testing is unavailable or limited and in such settings, similar to our study setting, CTX prophylaxis is required to continue uninterrupted for a longer period. Other South African studies concur, that less than 50% of HIV exposed children are tested before 6 months of age which suggests that more than half of HIV exposed children who initiate CTX prophylaxis will need to continue until 15 or 18 months of age when HIV status is confirmed by serological methods [16], [17].

The high antenatal HIV prevalence in the KwaZulu Natal province (40%) is suggestive that more than a third of children born in this province should receive CTX prophylaxis from 6 weeks until 15 months if more women intend breastfeeding for a longer period and if earlier infant diagnosis remains inaccessible. It is not surprising that the current practice of CTX prophylaxis in this province is unsatisfactory since CTX prophylaxis is only one of many interventions constituting a package of comprehensive care to ensure HIV-free survival among HIV exposed children [18]. And as expected, other interventions such as antiretroviral prophylaxis to prevent HIV transmission would take precedence in implementation, monitoring and evaluation at facility, district and provincial levels.

The primary purpose of CTX prophylaxis in all HIV exposed children is to afford added protection against other opportunistic infections in infants who are HIV infected and who would only be diagnosed much later. This is assuming that MTCT rates remain high and early diagnosis is not possible. One would argue, that with recent advances in maximizing PMTCT intervention efficacy with more complex and intensive antiretroviral regimens, only a fraction of HIV exposed children receiving CTX prophylaxis will potentially benefit from CTX since MTCT rates have been remarkably reduced. The current South African PMTCT policy (2010) provides for a longer ante partum ART prophylaxis (AZT from 14 weeks in pregnancy) in addition to the sdNVP in labour for women with a CD4>350. Women with a CD4<350 are eligible for ART as life long treatment. The policy supports a longer breastfeeding period (6–12 months), does not provide for free replacement feeds but does include daily NVP prophylaxis to infants to reduce the risk of MTCT through breastfeeding [19]. Given the current transmission rate of 4% at 8 weeks of age in KwaZulu Natal, South Africa, 96% of HIV exposed children would be unnecessarily exposed to CTX. Even though, breastfeeding practice may be more common and for a longer duration as a result of the new WHO and SA PMTCT guidelines, HIV transmission during breastfeeding in this setting is likely to be minimized by the daily Nevirapine prophylaxis during breastfeeding [20].

A secondary analysis of the PEPI-Malawi study further demonstrated the potential benefit of CTX prophylaxis in reducing morbidity and hospital admissions among HIV exposed uninfected children between 6 and 15 months of age, independent of breastfeeding [21]. There are however no studies seeking evidence of sustained protection of CTX in HIV exposed and uninfected children beyond the breastfeeding period. Some may argue that such studies are no longer valuable since CTX protection is most needed in children younger than 12 months and with the recent WHO Infant feeding recommendation, women will be breastfeeding for longer than 12 months. It is highly presumptive that majority of women in resource limited settings will be breastfeeding for 12 months or longer and as shown in our evaluation, breastfeeding practice is relatively minimal although the majority of women may not have met the AFASS criteria (data not included in this analysis) and yet chose not to breastfeed. Infant feeding choices may not always be the safest and the most desired and are often influenced by other social factors. Hence, further evidence for extended CTX prophylaxis in HIV exposed, uninfected children even during the non-breastfeeding period, is needed.

Though stock outs of other drugs are known to occur, the data in our study suggest that CTX was always available. Provision of CTX prophylaxis in this study population appears to be largely dependant on the lack of understanding of guidelines by the health service providers. Incorrect dosing by the mothers indicated that the women did not receive adequate information from the health care workers. Considering that half the women in the study population did not dose their infants over weekends, may reflect the implementation of older guidelines that prescribed CTX for 5 days a week. Training curricula for health care providers should incorporate CTX prophylaxis as an integral component of the comprehensive package of care for HIV exposed children. Dosing schedules, time to initiate and criteria for discontinuation should receive particular attention in health care worker training as well as in information sessions with patients.

As with any drug use, drug efficacy is directly proportional to the patient's level of adherence. This is true for CTX prophylaxis as well. Exposing children to CTX for a longer period is more likely to affect adherence to CTX. Daily dosing of cotrimoxazole in HIV infected children in a South African study was associated with a 2-fold lower incidence of bacteremia and shorter duration of hospital admission as compared to children receiving interrupted prophylaxis [22]. Although not statistically significant, intermittent cotrimoxazole prophylaxis in this South African study population was also associated with a higher prevalence of antibiotic resistance. Furthermore, poor adherence may not directly influence CTX resistance, but there is a potential for increased resistance to other antibiotics and as Gill et al have concluded that the benefit of CTX in HIV infected children would have to be weighed against the widespread antimicrobial resistance to a limited availability of low-cost antibiotics in resource-limited settings [23].

One of the limitations in our study is the selection bias of the study population. Only children whose mother's HIV status was known, were selected for study, hence our findings are more likely an over estimate. The proportion of HIV exposed children in the general population initiating CTX is expected be much lower considering the reported high attrition rates in PMTCT program follow-up, as well as lack of health documentation reporting the HIV status of the child as HIV exposed or unexposed. Secondly, being a retrospective cohort study our findings were mainly dependent on the available documentation and mother's memory recall.

In conclusion, the provision of CTX prophylaxis to HIV exposed children in a resource-limited setting with a high antenatal HIV prevalence is inadequate. Breastfed children, although a small proportion of the study population, were twice more likely to commence CTX at 6 weeks as compared to their non-breastfed counterparts. Considering changes in the infant feeding policy in South Africa and other resource limited settings, CTX coverage could be positively influenced in the future but still requires monitoring. Further research is warranted to determine the safety and effectiveness of continued CTX in confirmed HIV uninfected infants after cessation of breastfeeding.
